# Associations between subjective and objective measures of stress and load: an insight from 45-week prospective study in 189 elite athletes

**DOI:** 10.3389/fpsyg.2024.1521290

**Published:** 2025-01-21

**Authors:** Kristina Drole, Mojca Doupona, Kathrin Steffen, Aleš Jerin, Armin Paravlic

**Affiliations:** ^1^Faculty of Sport, University of Ljubljana, Ljubljana, Slovenia; ^2^Oslo Sports Trauma Research Center, Norwegian School of Sport Sciences, Oslo, Norway; ^3^Norwegian National Unit for Sensory Loss and Mental Health, Oslo University Hospital, Oslo, Norway; ^4^Institute of Clinical Chemistry and Biochemistry, University Medical Centre Ljubljana, Ljubljana, Slovenia; ^5^Faculty of Pharmacy, University of Ljubljana, Ljubljana, Slovenia; ^6^Faculty of Sports Studies, Masaryk University, Brno, Czechia

**Keywords:** stress, subjective perception, training load, academic load, weekly athlete monitoring, cortisol

## Abstract

**Introduction:**

The aim of this study was to investigate the associations between subjective and objective measures of stress and load in elite male handball players at both the group and individual levels.

**Methods:**

In this 45-week prospective cohort study, 189 elite male handball players weekly reported their perceived stress and load across training, competition, academic, and work domains. Blood samples were collected five times during the 2022/23 season to measure cortisol and the free testosterone to cortisol ratio (FTCR). We derived a “load” variable as the sum of training, competition, academic and work hours and calculated acute, chronic, and acute-to-chronic ratio variables for both load and stress. Associations between subjective and objective measures were analyzed using Spearman’s rank correlation.

**Results:**

Weak to moderate positive associations were found between load and perceived stress (*r* = 0.19 to 0.46, *p* < 0.001), and between perceived stress and cortisol (*r* = 0.10, *p* = 0.023). Weak negative associations were found between perceived stress and FTCR (*r* = −0.18 to −0.20, *p* < 0.001) and between load and FTCR (*r* = −0.13, *p* = 0.003). A total of 86% of athletes had positive associations between stress and load (47% weak, 34% moderate, 5% high); 78% between stress and cortisol (27% weak, 22% moderate, 29% high); and 63% demonstrated negative associations between FTCR and load (18% weak, 32% moderate, 13% high).

**Conclusion:**

This study highlights the complexity between subjective and objective measures of stress and load in athletes. Understanding the link between these measures may help coaches and sports scientists streamline athlete monitoring. In cases where moderate to strong associations exist, subjective measures might serve as a reliable substitute for objective ones, making the monitoring process more time- and cost-efficient.

## 1 Introduction

Athletes operate within a complex interaction of physical, psychological, and environmental stressors, all of which impact their performance, well-being, and health. Among the various factors influencing performance and health status, stress and load play crucial roles, exerting direct and indirect effects on athletes’ physiological and psychological states (Drole et al., 2023; Hamlin et al., 2019).

In order to optimize performance, prevent overreaching and potential health problems, athlete monitoring is incorporated into daily sports practice (Foster, 1998; Halson, 2014; Soligard et al., 2016). Effective athlete monitoring involves assessing both external and internal loads through subjective and objective measures to achieve a comprehensive understanding of athletes’ well-being (Soligard et al., 2016). Subjective measures, such as athletes’ self-reported perceptions of stress, fatigue, and mood, provide valuable information about their subjective experiences and mental states. Objective measures, including biochemical markers and training load metrics, offer complementary insights into athletes’ physiological responses and adaptation processes. Furthermore, devices such as wrist-worn monitors, smart watches, and chest straps can complement athlete monitoring with continuous, non-invasive measurements of physiological parameters like heart rate, sleep quality, skin temperature and movement patterns. By combining these measures, athlete monitoring programs can identify potential health issues early, mitigate injury/illness risks, optimize training loads, and enhance performance outcomes (Foster, 1998; Schwellnus et al., 2016).

However, certain objective measures, such as salivary or blood-derived biomarkers, are expensive and often unavailable for everyday use. Therefore, there is a need for time- and cost-effective methods for monitoring athletes’ responses, making it essential to understand the relationship between subjective and objective measures of stress and load (Saw et al., 2016). This understanding could help coaches and sports scientists streamline their monitoring processes, by relying more on accessible and affordable subjective measures without compromising accuracy.

Previous research has highlighted the associations between subjective measures, such as psychological questionnaires, and various objective markers, including cortisol levels (Jürimäe et al., 2004), cytokines (Main et al., 2009), heart rate variability (Immanuel et al., 2023; Miyatsu et al., 2022), and training load parameters (Umeda et al., 2008). A recent systematic review of 56 studies has shown that subjective measures are more sensitive and consistent than objective measures in determining response to both acute and chronic training loads (Saw et al., 2016). However, the authors could not confirm consistent associations between subjective and objective measures, due to methodological heterogeneity, such as differing measures, sampling frequencies, and statistical methods used in original studies (Saw et al., 2016). Moreover, none of the studies included in this review (Saw et al., 2016) or the published literature accounted for non-sport-related stressors when investigating associations between subjective and objective measures. Many athletes pursue an educational or professional path outside of their athletic career (i.e., dual career), which adds additional loads and stressors to their sports-related load (Drole et al., 2023). Incorporating these stressors, particularly those related to dual careers, is crucial, as balancing academic or professional responsibilities with training can lead to mental and physical fatigue, influencing both subjective perceptions of stress and objective physiological markers (Hamlin et al., 2019; Lopes Dos Santos et al., 2020; Mann et al., 2016; Pimenta et al., 2021).

Therefore, our study aimed to: (i) investigate the associations between subjective and objective measures of stress and load in athletes. Specifically, we investigated how athletes’ perceptions of stress are associated with physiological biomarkers of stress such as cortisol levels and free testosterone to cortisol ratio (FTCR), as well as with objective measures of load, such as sport-specific, academic and work load. Secondly, we aimed to (ii) investigate the individuals’ associations between selected subjective and objective measures to determine the percentage of athletes with weak, moderate, or high associations, thereby identifying the proportion of athletes in which subjective reporting align well with objective measures.

## 2 Materials and methods

### 2.1 Study design and participants

This study was designed as a prospective cohort study and was conducted in accordance with the Strengthening the Reporting of Observational Studies in Epidemiology (STROBE) (Cuschieri, 2019) guidelines and A CHecklist for statistical Assessment of Medical Papers (CHAMP) (Mansournia et al., 2021). The sample size was calculated prospectively based on predefined parameters and is detailed in the previously published study protocol (Drole et al., 2023).

We invited first men handball league players (Tier 4: Elite level) (McKay et al., 2022) to participate in the study and screened them with the following inclusion criteria: male handball players above 18 years of age, who are competing in the first Slovenian handball league. If they met the criteria and agreed to participate, athletes and team staff were informed about the study purpose and asked to sign the participation consent. We conducted the study in accordance with the latest version of the Declaration of Helsinki and approved by the National Medical Ethics Committee of Slovenia (number: 0120-109/2022/3). The study was prospectively registered on Clinical Trials.gov (registration number: NCT05471297).

### 2.2 Materials and procedure

**FIGURE 1:**
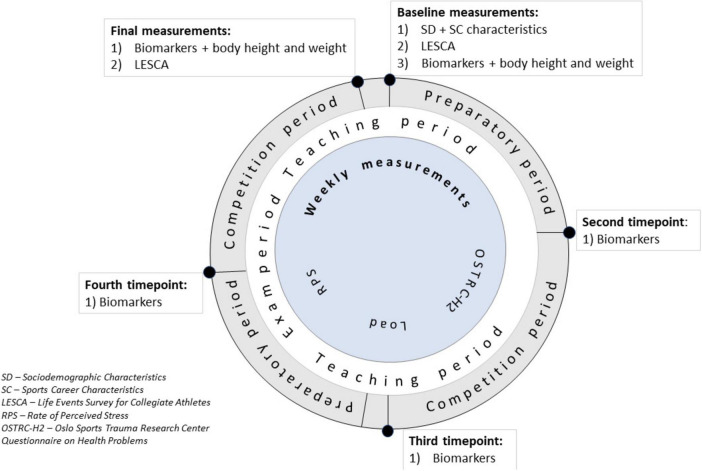
Assessment plan. Adapted and reused with permission from Drole et al. (2023), originally published in BMJ Open, 2023. doi: 10.1136/bmjopen-2022-069104.

We followed the athletes through 45 weeks between July 19th 2022 and June 2nd 2023 during the entire 2022/23 handball season, according to the previously published protocol (Figure 1) (Drole et al., 2023).

#### 2.2.1 Subjective measures

Athletes weekly reported their perceived stress on a 10-point scale (1 = no stress, 10 = extreme stress), which we defined as a subjective measure of stress (Lesage et al., 2012). This utilization of a single question to rate perceived stress on a visual analogue scale (VAS), instead of several long questionnaires is widely used in clinical setting, and was recommended for research as well (Mitchell et al., 2008). Previous studies have confirmed good validity (Cox and Davison, 2005), sensitivity (Lesage et al., 2012), high inter-rater reliability (Berjot et al., 2011; Lesage and Berjot, 2011) and associations between the stress VAS and other tools to assess stress (Cohen et al., 1983; Lesage and Berjot, 2011).

#### 2.2.2 Objective measures

##### 2.2.2.1 Load

Together with their support staff (coaches, strength & conditioning coaches and physiotherapists), the athletes reported weekly loads (in hours) in four domains:

(I) Training load: was segmented into sport-specific (handball) training and strength & conditioning training;
(II) Competition load: measured in minutes of games played, which were converted into hours for the purpose of statistical analysis;
(III) Academic load: lectures, exams, practical courses and study hours;
(IV) Work load: additional employment alongside athletic career.

A new variable, “load” was derived as a sum of training, competition, academic and work load and expressed in hours.

Moreover, acute (A), chronic (C) and acute to chronic ratio (ACR) were calculated for both load and perceived stress. The acute variable was accounted for the value of the measurement week, while the chronic variable was averaged over the week of measurement and the three preceding weeks. Additionally, we determined the ACR by the following formula, where A is the “acute workload” (workload carried out in the last 7 days) and W1, W2 and W3 are the workloads carried out in the previous 3 weeks: (Gabbett et al., 2016; Murray et al., 2017)


$$A\,C\,R=\frac{A}{0.25\times \left(W\,1+W\,2+W\,3+A\right)}$$

ACR for perceived stress was calculated using the same formula.

##### 2.2.2.2 Blood biomarkers

Blood samples were collected five times throughout the 2022/23 season (before/after: preparatory period, competition period, off-season period). All samples were taken between 07:00 and 9:00 in the morning, in a quiet room with an optimal temperature setting at handball facilities. Cortisol and FTCR were defined as objective measures of stress and load, respectively. Athletes were instructed to avoid strenuous exercise the day before. Two blood samples were drawn from the antecubital vein using BD Vacutainer^®^ SST II Advance Tubes containing polymer gel, by an experienced nurse while the subjects were seated. The samples were then centrifuged for 10 min at 1,500×*g* (Tehtnica, Centric 160). Post-centrifugation, the samples were placed in specialized containers to maintain the appropriate temperature and transported to a laboratory for analysis. Cortisol levels in the serum were measured using an electrochemiluminescence assay (Cobas e411 analyzer, Roche Diagnostics, Mannheim, Germany), with a detection limit of 0.5 nmol/L. Free testosterone was calculated from testosterone, SHBG, and albumin using the Vermeulen equation (Vermeulen et al., 1999). Serum testosterone and SHBG were also measured with an electrochemiluminescence assay (Cobas e411 analyzer, Roche Diagnostics, Mannheim, Germany), with detection limits of 0.09 and 0.35 nmol/L, respectively. Albumin levels in the serum samples were measured spectrophotometrically using bromocresol green (Alinity analyzer, Abbott Laboratories, Illinois, USA), with a detection limit of 10 g/L.

### 2.3 Statistical analysis

We conducted the statistical analysis using the SPSS software (version 29.0, IBM Inc, Chicago, United States of America). Descriptive statistics were used to summarize demographic characteristics of subjects and outcomes of interest, and are presented as mean ± standard deviation. To enable individual comparisons, we normalized each player’s load to their maximum value and used this value for all further analyses involving the “load” variable. Next, the normality of data distribution using the Shapiro–Wilk test was tested. Due to the non-normal distribution of most of the data of interest we used the Spearman’s rank correlation to investigate the associations between subjective and objective measures of load and stress. This included examining the associations between load (A, C, ACR) and perceived stress (A, C, ACR), load (A, C, ACR) and cortisol levels, load (A, C, ACR) and the FTCR, and perceived stress (A, C, ACR) and cortisol levels. Further, we conducted sub-analyses at the individual level to determine the percentage of athletes with weak, moderate, or high associations between selected subjective and objective measures of stress and load. The following thresholds of the correlation coefficient were used to assess magnitude of the relationships analyzed: weak ≤ 0.35; 0.36 ≤ moderate < 0.67; 0.68 ≤ high < 1 (Taylor, 1990). Statistical significance for all analyses was accepted at *p* ≤ 0.05.

## 3 Results

Initially, we enrolled 189 athletes in the 45-week prospective study. After accounting for dropouts, and considering that two and six teams, respectively, started the season 1 and 2 weeks later, we included a total of 7,946 observations involving all measures of interest (perceived stress, load, and biomarkers) in the final analysis. The overall response rate was 91%. The athletes’ characteristics, playing history and load exposure are presented in Table 1. Terms in Table 1, such as “Weekly Training Load” and “Perceived Stress,” are explained in detail in the Methods section under “2.2.1 Subjective measures” and “2.2.2 Objective measures.”

**TABLE 1:** Participants’ characteristics.

PARTICIPANTS’ CHARACTERISTICS (*N* = 189)	
Age (years)	23.3 ± 4.4
Height (cm)	188.9 ± 6.3
Weight (kg)	92.4 ± 11.3
BMI (kg/m^2^)	25.9 ± 2.3
Handball experience (years)	13.8 ± 4.4
Weekly training load (hours)	8.6 ± 4.4
Weekly competition load (hours)	0.3 ± 0.4
Weekly academic load (hours)	3.7 ± 7.6
Weekly work load (hours)	5.2 ± 12.3
Perceived stress	4.8 ± 1.8

n, number of athletes; BMI, body mass index; data presented as mean ± standard deviation.

### 3.1 Associations between perceived stress, load and biomarkers

**FIGURE 2:**
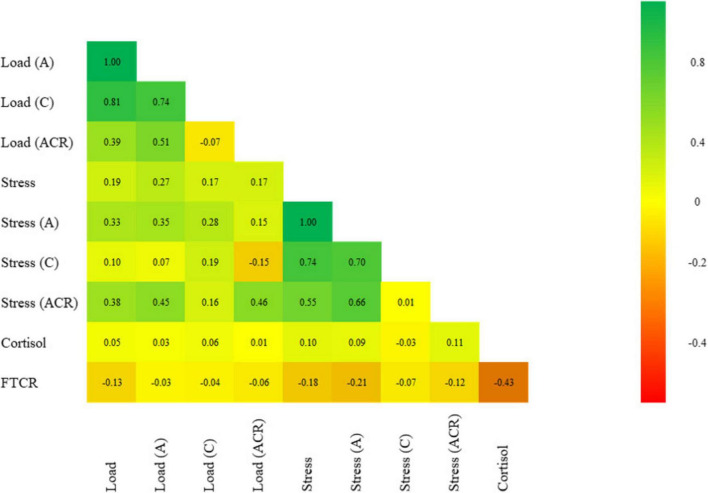
Subjective - objective measures associations heat map. The color intensity represents the strength of the correlations, with darker hues indicating stronger associations. All the associations are significant (*p* < 0.05). This visualization provides insight into the associations between subjective and objective measures of stress and load.

Figure 2 shows the association between different measures of load and stress. Weak to moderate associations were observed between load and perceived stress (weak, *r* = 0.19, *p* < 0.001), load and acute perceived stress (weak, *r* = 0.33, *p* < 0.001), acute load and acute perceived stress (weak, *r* = 0.35, *p* < 0.001), and load ACR and perceived stress ACR (moderate, *r* = 0.46, *p* < 0.001). Weak associations were observed between perceived stress and biomarkers. Specifically, perceived stress had a weak positive association with cortisol (*r* = 0.10, *p* = 0.023), and a weak negative association with FTCR (*r* = −0.18, *p* < 0.001). Similarly, acute perceived stress showed a weak negative association with FTCR (*r* = −0.21, *p* < 0.001), whereas perceived stress ACR had a weak positive association with cortisol (*r* = 0.11, *p* = 0.036). Additionally, there was a weak negative association between load and FTCR (*r* = −0.13, *p* = 0.003) (Figure 2).

### 3.2 Individual associations between perceived stress, load and biomarkers

**FIGURE 3:**
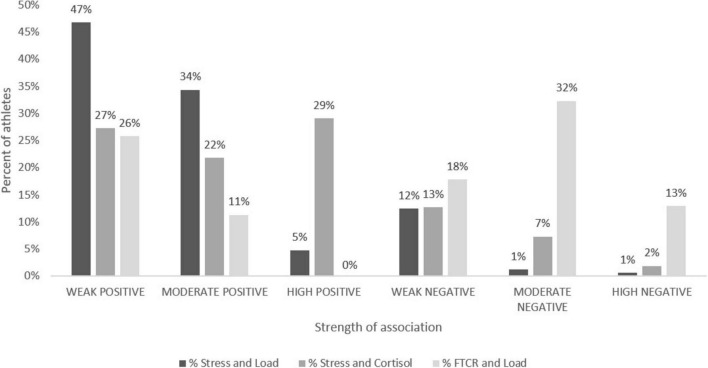
Athletes divided into groups by strength of associations between selected subjective and objective measures. The groups (e.g., high, moderate, and weak associations) highlight variability in how subjective measures align with objective measures. Key measures include perceived stress, load, cortisol and free testosterone to cortisol ratio (FTCR).

The results showed varying percentages of individuals whose measures were weakly, moderately, or highly associated; both positively and negatively (Figure 3). When perceived stress and load were considered, 47, 34, and 5% of athletes had weak, moderate, and high positive associations, respectively, whereas 12, 1, and 1% of athletes had weak, moderate, and high negative associations, respectively. For the association between perceived stress and cortisol, 27, 22, and 29% of athletes showed weak, moderate, and high positive associations, respectively, while 13, 7, and 2% exhibited weak, moderate, and strong negative associations, respectively. Similarly, for the association between load and FTCR, 26 and 11% of athletes showed weak and moderate positive associations, whereas 18, 32, and 13% demonstrated weak, moderate, and high negative associations (Figure 3).

## 4 Discussion

The main aim of this prospective cohort study was to investigate the associations between subjective and objective measures of stress (VAS, cortisol), FTCR and sport-, academic- and work-load in Slovenian elite male handball players on group level and individual basis. From the practical perspective, we aimed to provide guidance to all handball practitioners whether rather costly and time-consuming invasive measures of blood biomarkers could be replaced with subjective measures of athletes’ well-being.

To the best of our knowledge, this is the first study to take holistic approach in defining load by including athletes’ non-sports-related loads and examining these associations in a large sample representing the first men’s handball league. Our findings revealed weak to moderate associations between subjective and objective measures of stress and load when analyses were conducted on the entire sample, and high variability among individuals.

### 4.1 Load and stress

Monitoring athletes’ internal training loads in response to external loads is crucial for assessing whether they are effectively adapting to their training program. We observed weak to moderate positive associations between load and perceived stress. The strongest relationship (*moderate*, *r* = 0.46) was identified between the load ACR and the perceived stress ACR. This suggests that fluctuations in load over time and accumulation of load are mirrored by changes in acute perceived stress levels, highlighting the importance of considering both short-term and long-term load management in training programs. Our results align with findings from a systematic review on 56 studies investigating athletes of various ages and levels of competition that show impaired well-being with increased training load and vice versa, confirming that subjective measures respond in a dose-dependent manner to training load (Saw et al., 2016). Several previous studies have shown the influence of training load on stress (Bok et al., 2022; Nobari et al., 2020; Nobari et al., 2023). Fullagar et al. (2017) found that well-being variables such as sleep quality, delayed onset of muscle soreness (DOMS), energy level and overall wellness were at their lowest the day after a rugby league or American football match and did not recover to baseline levels for at least 4 days. Another study demonstrated that the training loads performed in the previous week strongly affected rates of perceived exertion, with medium ratings of perceived exertion providing the highest predictive accuracy for subsequent training outcomes (Rossi et al., 2019). Furthermore, Nobari et al. (2020) observed the highest values of fatigue, DOMS and stress at the end of the season, and the lowest values of sleep and stress in the early season, indicating the chronic effect of training and competition load on well-being parameters. Recent research also suggests that high training loads, without adequate recovery, can lead to chronic stress and increased risk of burnout, which supports the need for comprehensive load management strategies (Brenner and Watson, 2024; Malone et al., 2018).

Additionally, stress from non-training-related sources, such as work or social life, and the balance between training and recovery, can exacerbate training-related stressors (Jeffreys, 2005). Our study extends these findings by also considering non-training-related stressors, such as academic and work-related loads, recognizing the significant impact of total load on athlete’s perceived stress. This comprehensive approach provides a more holistic understanding of the various factors influencing athletes’ well-being and underscores the importance of integrating both training and non-training stressors in stress and load monitoring systems.

### 4.2 Stress and biomarkers

Compared to the consensus of a recent systematic review on the association between stress and cortisol in athletes (Saw et al., 2016), our study revealed a significant but weak-to-moderate positive association between perceived stress and cortisol levels. This finding indicates that higher cortisol levels correspond to athletes’ heightened perceptions of stress, which aligns with existing literature identifying cortisol as a key biomarker of physiological stress response (Russell and Lightman, 2019). However, it is important to acknowledge that stress perception is influenced by range of psychological and contextual factors, meaning each athlete responds to stress differently. Consequently, the variability in associations observed among athletes may reflect that elevated cortisol levels do not consistently correspond to heightened stress perception in every individual. In the context of athletes, cortisol levels can rise significantly during periods of intense physical activity and psychological stress, making it a reliable indicator of overall stress levels (Kraemer and Ratamess, 2005). Elevated cortisol levels not only reflect acute stress responses but also chronic stress exposure, which in the next step can impair recovery and performance (Arnsten, 2009; Russell and Lightman, 2019). Moreover, the positive association between perceived stress and cortisol levels suggests that athletes’ subjective experiences of stress are linked with physiological adaptations.

Conversely, we found negative associations between perceived stress and FTCR. A lower FTCR, indicative of higher cortisol levels and/or lower testosterone levels, was most pronounced for the acute perceived stress variable. This association suggests that the hormonal imbalance reflected in the FTCR can significantly impact athletes’ perceptions of stress in the present moment. Previous studies have demonstrated that acute cortisol responses increase with substantial training stress, indicating a direct association between training load and cortisol levels (Häkkinen and Pakarinen, 1993; Kraemer et al., 1993). Consequently, during periods of high training stress, the body’s catabolic processes may dominate, leading to elevated cortisol levels (Fry et al., 1991). The (free) testosterone to cortisol ratio has been proposed as a potential marker for the anabolic/catabolic status of athletes, with a lower ratio indicating increased stress and catabolism (Adlercreutz et al., 1986). Elevated cortisol during periods of increased load can disrupt the anabolic/catabolic balance, exacerbating stress perceptions and potentially leading to symptoms of overtraining.

### 4.3 Load and biomarkers

In addition to the stress-related findings, we observed a negative association between load and the FTCR. Higher loads were associated with decreased FTCR, further supporting the notion that intense physical and psychological exertion can alter hormonal balance in ways that might affect both performance and recovery (Drole et al., 2024). This finding is consistent with the existing literature, where decreased FTCRs were observed with overreaching in several sports, such as rowing (Vervoorn et al., 1991), cycling (Hoogeveen and Zonderland, 1996), handball (Drole et al., 2024), and rugby (Maso et al., 2004). This relationship underscores the need for careful monitoring and management of training loads to maintain optimal hormonal balance and performance.

### 4.4 Variability in individual-level associations between subjective and objective measures of stress and load

With analyses on the individual level, we aimed to determine the percentage of athletes with weak, moderate, or high associations, thereby identifying the proportion of athletes in which subjective reporting aligns well with objective measures. Additionally, we aimed to estimate the number of athletes for whom subjective measures alone might be sufficient. We found high variability, indicating athletes have varying magnitudes and directions of associations between selected subjective and objective measures. This variability underscores the necessity for personalized training and stress management strategies that consider individual physiological and psychological profiles. The variabilities observed in the associations between subjective and objective measures of stress and load can be attributed to various biological and psychological factors. Biological variations, such as fluctuations in hormone levels, play a significant role. For example, individual differences in cortisol and testosterone concentrations can influence stress responses and recovery rates. Hormonal fluctuations are not uniform across individual athletes and can be affected by numerous factors including genetics, age, sex, lifestyle beyond sport, and overall health (Cappola et al., 2023). Genetic predispositions, such as polymorphisms in genes related to stress regulation may modulate the body’s hormonal or neural response to stress. Catechol-O-methyltransferase (COMT) rs4680 and brain-derived neurotrophic factor (BDNF) rs6265 are two of the most extensively studied single-nucleotide polymorphisms associated with stress responses (Armbruster et al., 2012; Chen et al., 2004; Cotman and Berchtold, 2002; Kang et al., 2013; Kim et al., 2010; Shalev et al., 2009). Increases in BDNF promote neurogenesis, resilience to brain damage, and improved cognitive function (Cotman and Berchtold, 2002). The COMT Val158Met (rs4680) polymorphism affects dopamine availability in the prefrontal cortex by increasing enzymatic activity (Chen et al., 2004). The BDNF Val66Met polymorphism has been shown to interact with recent life stress and influences hypothalamic–pituitary–adrenal (HPA) axis reactivity to psychological stress (Kim et al., 2010; Shalev et al., 2009). Beyond genetics, lifestyle factors such as poor sleep and sleep deprivation are major sources of psychological and physiological stress (de Almondes et al., 2021; Lo Martire et al., 2024; Meerlo et al., 2008; Van Laethem et al., 2015). Dietary habits, alcohol or caffeine consumption can further impact stress physiology and recovery capacity. Although moderate caffeine consumption has several beneficial acute effects, such as enhanced mood, alertness (Ferré, 2008; Lorist and Tops, 2003) and improved exercise performance (Guest et al., 2021), a substance methylxanthine also serves as an antagonist of adenosine receptors, boosting brain energy metabolism while reducing cerebral blood flow. This causes hypoperfusion, activation of norepinephrine neurons, and affects dopamine release, contributing to heightened stress and anxiety (Nehlig et al., 1992; Nehlig, 2016). Additionally, environmental factors such as seasonal variations, travel-related stress, or disruptions in circadian rhythms (e.g., jet lag) may confound the relationship between subjective and objective measures. On the other hand, psychological factors, including individual perceptions of stress, also contribute to these variabilities. Perception of stress is highly subjective and can vary widely among athletes. Factors such as past experiences, mental health, coping mechanisms, and support systems all shape how an individual perceives and responds to stress (Cohen and Wills, 1985; Wang et al., 2021). For instance, athletes with higher resilience or better coping strategies may perceive the same level of stress as less taxing compared to those with less effective coping mechanisms (Palamarchuk and Vaillancourt, 2021; Yalcin-Siedentopf et al., 2021). Various other factors such as education, age or training experience can moderate this effect as well. Another interesting observation was found as a result of our study, that is, either small or large samples can blur the actual associations. Therefore, although we aim to have large, representative samples, the associations should always be investigated within the specific population of interest and considered in the context of clinical settings and practice.

Although we confirmed high variability between individuals, we found that in the majority of individuals subjective measures are aligned well with objective measures. If we only consider moderate and high associations, the objective measures could be replaced with subjective measures in approximately ½ of the athletes. Thus, in general, the proposed measures are useful for monitoring stress and load in athletes. In the real-world setting, sports practice usually deals with smaller samples, such as teams or individuals. Therefore, understanding how each team and individual perceives his/her stressors, is crucial. This could be achieved by monitoring the athletes over a period of time using both subjective and objective measures to determine which athletes have high associations between their perceived stress, load and biochemical markers. When those individuals are identified, coaches and sport scientists should decide whether some measures can be replaced with only subjective reporting, as this approach not only reduces the financial and logistical burden associated with frequent biomarker testing, but also empowers athletes to be more involved in their own health monitoring. With establishing associations between subjective and objective measures, coaches and sports scientists can gain confidence in using tools such as self-reported stress scales and perceived exertion ratings to assess athletes’ well-being. Moreover, personalized stress and load monitoring, based on each athlete’s individual responses, can lead to more precise and timely adjustments in training programs. This ensures optimal performance while minimizing the risk of overtraining, injury, and illness.

Stress and load, though often defined separately, are intrinsically interconnected, representing facets of the same underlying construct. For example, training load can pose stress to an athlete, but stress can exist even without this specific load. Training load, for instance, imposes physical and psychological stress on an athlete, influencing their performance and recovery. This is reflected in the body’s physiological response, where elevated training loads can increase cortisol levels, while optimal training load can result in testosterone level increase. However, stress is not limited only to physical exertion. Athletes may experience stress from various sources such as competition load, which includes the pressures and demands of participating in competitive events, and academic load, where balancing educational responsibilities adds to their overall stress burden (Hamlin et al., 2019). Workload, encompassing professional or part-time job demands, and psychosocial stressors, including interpersonal relationships and social dynamics, further contribute to an athlete’s stress profile. For example, high cortisol levels not only reflect the body’s immediate reaction to physical training but also indicate chronic stress from continuous academic pressures or social interactions beyond the court. Several internal stressors, such as poor physical conditioning, performance expectations, injury and rivalry (Garvican-Lewis et al., 2018), and external stressors, such as sport organizations, including relationships, interpersonal demands, training environment (Mellalieu et al., 2009; Pensgaard and Roberts, 2000), and coaching-related stressors (Gearity and Murray, 2011; Isoard-Gautheur et al., 2012; Kimball and Freysinger, 2003) have been associated with elite sport. Moreover, insufficient support networks, time-management, travel, fatigue due to lack of sleep and transition to tertiary education or higher level of competition have been reported to be a source of stress (Hanton et al., 2005). These diverse loads can cumulatively impact the perception of stress, leading to varying physiological responses. Recognizing the multifaceted nature of stress and load underscores the importance of a holistic approach in managing athlete well-being, where both physical training and external stressors are considered to optimize performance and health (West et al., 2021).

### 4.5 Clinical implications

Certain objective measures, like salivary or blood-derived biomarkers, are costly, time consuming and impractical for daily use in athlete monitoring, which underscores the need for better alternatives. By determining the associations between objective and subjective measures, this study highlights the potential for coaches and scientists to find opportunities to simplify monitoring processes, potentially shifting toward primarily using subjective reporting while maintaining accurate and effective stress and load management.

### 4.6 Limitations

In this study, we included the largest sample of athletes investigated for blood biomarkers to date, featured weekly follow-ups and achieved a high response rate from athletes. The study’s strengths include collecting three to five blood samples from each athlete, which enhances the robustness and reliability of the data. However, the reliance on male handball athletes limits the generalizability of the findings to other sports and female athletes. Although large homogeneous samples are beneficial for interpreting results, particularly in hormone measurements, future research should explore these associations within diverse populations, including varying sexes, ages, sports, levels of competition, and social backgrounds. Additionally, the use of a single question to measure perceived stress (the 10-point visual analog scale) may limit the sensitivity of the stress assessment. While this simple and practical measure allows for easy tracking of perceived stress over time in large cohorts like ours, it may not fully capture the complexity and multidimensional nature of stress. The reliance on a single-item measure may overlook subtle variations in stress experiences across individuals, and more comprehensive or multi-dimensional stress assessments could provide a richer understanding. Future studies should aim to identify factors that differentiate the individuals with high, moderate, weak, or non-existent associations between subjective and objective measures. Furthermore, the integration of wearable technologies in the research examining the relationship between subjective and objective measures of stress and load warrants future investigation. Moreover, our study was not designed to explore cause-and-effect relationships between the measures of interest. Therefore, future research should address this question using alternative study designs and methodologies to provide practitioners with a deeper understanding of this topic. Beyond large-scale studies, it is essential for coaches and sports scientists to assess these associations within their own teams and/or individual athletes. Understanding the unique stress and load profiles of their athletes allows for more tailored interventions.

## 5 Conclusion

The study highlights the complexity of the associations between subjective and objective measures of stress and load in athletes. The findings reveal weak to moderate associations at the group level, with significant individual variability, emphasizing the need for personalized load and stress management strategies. While both subjective and objective measures remain valuable tools for monitoring stress and load, our results suggest that, for many athletes, particularly those with moderate to strong associations, subjective assessments may accurately reflect their physiological state. These insights not only enhance the understanding and monitoring of athlete well-being but also support the potential substitution of objective measures with subjective ones. This approach could simplify and streamline athlete monitoring processes, enhancing efficiency without compromising accuracy.

## Data Availability

The raw data supporting the conclusions of this article will be made available by the authors, without undue reservation.
